# How to Survive without Water: A Short Lesson on the Desiccation Tolerance of Budding Yeast

**DOI:** 10.3390/ijms25147514

**Published:** 2024-07-09

**Authors:** Zoe L. Robison, Qun Ren, Zhaojie Zhang

**Affiliations:** Department of Zoology and Physiology, University of Wyoming, Laramie, WY 82071, USA; zrobison@uwyo.edu (Z.L.R.); qren@uwyo.edu (Q.R.)

**Keywords:** anhydrobiotes, desiccation stress, desiccation tolerance, *Saccharomyces cerevisiae*, survival

## Abstract

Water is essential to all life on earth. It is a major component that makes up living organisms and plays a vital role in multiple biological processes. It provides a medium for chemical and enzymatic reactions in the cell and is a major player in osmoregulation and the maintenance of cell turgidity. Despite this, many organisms, called anhydrobiotes, are capable of surviving under extremely dehydrated conditions. Less is known about how anhydrobiotes adapt and survive under desiccation stress. Studies have shown that morphological and physiological changes occur in anhydrobiotes in response to desiccation stress. Certain disaccharides and proteins, including heat shock proteins, intrinsically disordered proteins, and hydrophilins, play important roles in the desiccation tolerance of anhydrobiotes. In this review, we summarize the recent findings of desiccation tolerance in the budding yeast *Saccharomyces cerevisiae*. We also propose that the yeast under desiccation could be used as a model to study neurodegenerative disorders.

## 1. Introduction

Water is the most abundant yet essential molecule in all living organisms on Earth, making up about 60–90% of the total mass in living organisms [1]. It is essential not only for life on Earth but even in the search for life on other planets; it serves as an important biotic indicator [2]. Water functions as a solvent and is required for many biological processes. When organisms do not have water, the consequences are detrimental. Certain organisms, however, can survive with minimal water for long periods of time. These organisms are commonly called anhydrobiotes, which are a group of unique organisms that can survive anhydrobiosis or life without water [3]. Understanding how anhydrobiotes respond and mitigate the stress caused by desiccation could help us better understand stress biology. This is particularly important as today’s climate change occurs more dramatically, and water resources become scarcer.

The capacity of resistance to desiccation is not limited to one species. Anhydrobiotes have been identified in bacteria, yeast, plants, and small animals, such as tardigrades, which can survive for years without water [3,4]. Plant seeds, for example, lose up to 95% of water during maturation and maintain their viability under harsh environmental conditions [5]. Tardigrades can not only survive near-complete desiccation but also other environmental extremes, such as a high vacuum, high/low temperatures, and UV radiation [6]. The budding yeast *Saccharomyces cerevisiae* can also survive desiccation and has been widely used in the industry of baking, brewing, and other biotech fields [7].

Desiccation imposes a variety of stresses on the organism, such as osmotic stress, oxidative stress, and DNA damage stress. These stresses are likely interconnected and provide cross-protection between different stresses. Transient heat shock, for example, significantly increases the desiccation tolerance of desiccation-sensitive yeast cells [8]. Other stresses, such as osmotic or oxidative stress, have less of an impact on desiccation tolerance, suggesting that cross-protection is not equal among different stresses. A similar gene expression profile is observed when budding yeast is imposed with different environmental stresses [9], further suggesting the interconnections among different stresses.

Anhydrobiotes, such as yeast, proceed through many preconditioning steps to acquire desiccation tolerance. During these preconditioning steps, they undergo a range of morphological and physiological changes, such as thickened cell walls and the accumulation of stress proteins to prepare themselves for desiccation tolerance [10,11]. In addition, cells have shown metabolic changes to promote survival, using food reservoirs and reducing metabolic activities. Certain metabolic pathways, such as the fatty acid oxidation pathway, become more apparent during the desiccation process [12]. Some metabolic processes could be halted during desiccation [13].

In this review, we briefly discuss recent findings of the desiccation stress and stress response of anhydrobiotes, specifically of the budding yeast *Saccharomyces cerevisiae*. We attempt to address two major questions: how do anhydrobiotes acquire desiccation tolerance, and how do anhydrobiotes maintain life during the desiccated state? In addition, we briefly discuss the feasibility of using desiccated yeast as a model to study prion diseases, which share certain common features with desiccated yeast, like protein misfolding [14].

## 2. How Anhydrobiotes Acquire Desiccation Tolerance

### 2.1. No Pain, No Gain—Low Stress Produces More Tolerance

Tolerance to oxidative stress is significantly enhanced when yeast cells are first exposed to low levels of oxidative stress [15]. Similarly, the desiccation tolerance of anhydrobiotes is not innate. They must proceed through a ‘painful’ process, i.e., experiencing a low level of stress in order to gain desiccation tolerance—the capacity to survive severe water loss. This stress may or may not be directly related to desiccation. In plants, for example, the acquisition of desiccation tolerance is triggered by gradual dehydration and an increase in the production of abscisic acid [16,17]. Nematodes (*Caenorhabditis elegans*) gain their desiccation tolerance by experiencing starvation, which induces dauer larvae, followed by initial dehydration at high relative humidity (preconditioning) [18,19]. Preconditioning is also required for tardigrades to gain desiccation tolerance via initial slow drying [20,21]. Desiccation tolerance in yeast is commonly induced via starvation by growing cells into the stationary phase when cells significantly reduce their metabolic level [22]. Under starvation, diploid yeast cells undergo meiosis and form spores, which are resistant to a wide range of harsh environmental stresses, such as UV radiation, varying temperatures, and desiccation [23].

**Figure 1:**
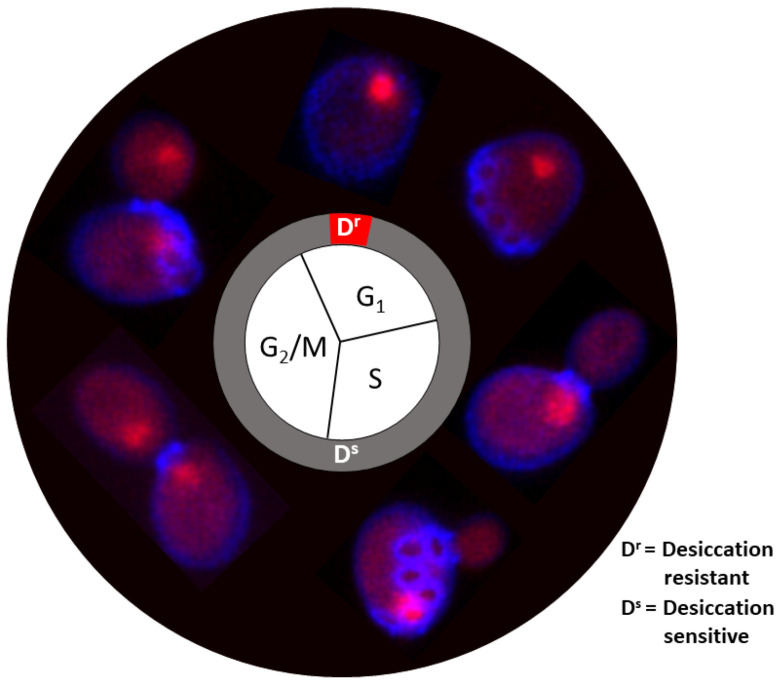
Cell cycle and desiccation tolerance in *S. cerevisiae*. Red: DRAQ5 staining of nuclei. Blue: fluorescence Brightener 28 staining of the cell wall and bud scars, which indicate the replicative age of yeast cells. The desiccation-resistant cells (D^r^) are in G_1_ but have higher fluorescence intensity. All other cells, including old G_1_ cells (multiple bud scars), and young or old G_2_/S/M cells, are desiccation sensitive (D^s^) (Redrawn based on [24]).

Compared to stationary (starved) cells, dividing yeast cells growing in a rich medium (log phase) have minimal tolerance to desiccation with a survival rate ranging from 0.0001% to 10%, depending on the desiccation method [12,22]. One intriguing question is as follows: what are these ‘lucky’ surviving cells, and why they are so ‘lucky’? Our recent study revealed that these ‘lucky’ cells share a few unique characteristics, including the following: (1) they are all replicative young cells; (2) they are all in the G1 phase of the cell cycle; and (3) they all have condensed chromosomes. The condensed chromosome suggests that genes are silenced, which is confirmed by the low metabolic activities of these cells [24] (Figure 1). One possibility is this could be a feature developed through evolution, in which a small portion of the cells is always prepared to encounter the unpredicted and sudden changes in their environment.

Cell cycle arrest has also been reported in both plants [25,26] and algae during desiccation [27]. DNA synthesis is repressed, and the new cycle of cell division is halted upon desiccation stress. The down-regulation of cell cycle-associated transcripts is observed in the desiccation-tolerant microalgae *Klebsormidium.* Under desiccated conditions, cells arrest at the G1 checkpoint, preventing entry into the S phase, where DNA replication occurs [27]. Desiccation causes DNA damage due to the formation of reactive oxygen species (ROS) and other stressors, so cell cycle arrest helps with coordinating DNA repair mechanisms [28]. The failure of the cell cycle arrest results in cell death [29,30]. These studies suggest that cell cycle and stress response pathways are interconnected, with many stress response genes being regulated in a cell cycle-dependent manner. For example, certain transcription factors involved in the stress response, like Msn2/4, are regulated by cell cycle-dependent mechanisms. These factors can activate the expression of genes encoding protective proteins, such as chaperones and antioxidants, which help cells cope with desiccation stress [31].

### 2.2. Morphological and Physiological Changes in Response to Desiccation Stress

#### 2.2.1. Morphological Changes

In response to stress, anhydrobiotes make significant morphological and physiological changes to prepare themselves for harsher conditions. In yeast, starvation stress leads to a thickened cell wall that is more thermotolerant and more resistant to enzymatic digestion [31]. Their metabolic level is also significantly reduced [32]. Stationary cells have more mitochondria because they have to use ethanol as their carbon source since glucose is depleted. Most of the stationary cells also accumulate more lipid droplets, which consist primarily of neutral lipids, triacylglycerols, and steryl esters, as a reservoir for energy [33]. When they are re-introduced into fresh nutrients, the lipids are quickly consumed before cells use external nutrients for cellular growth [34].

**Figure 2:**
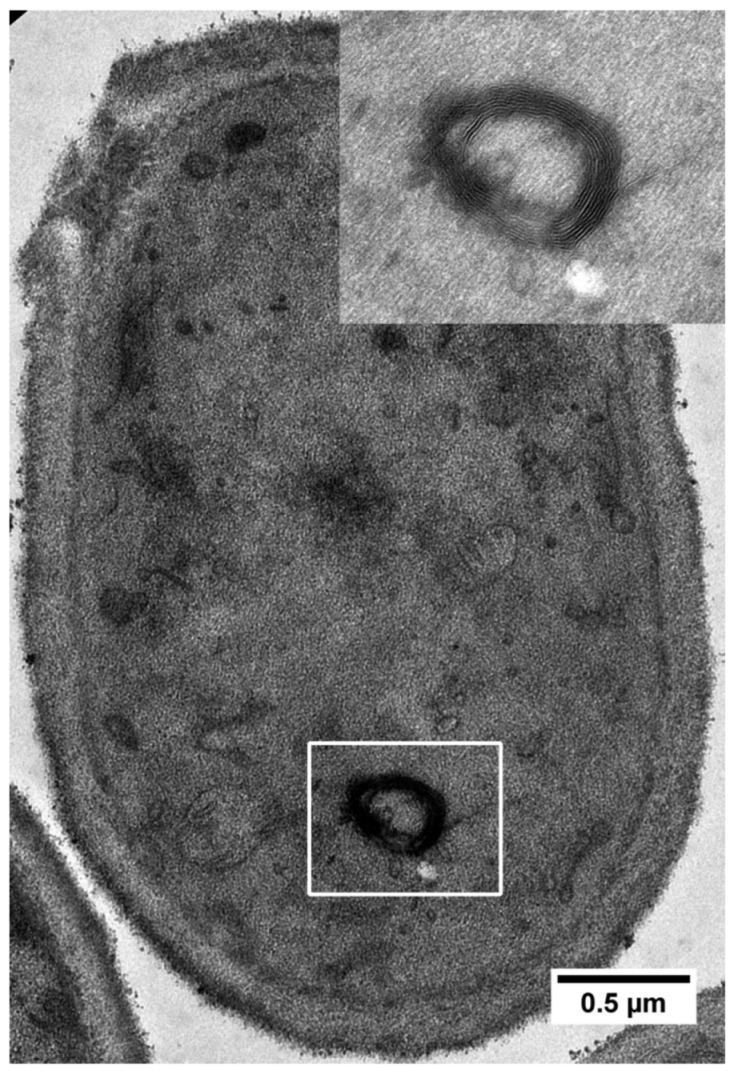
TEM image of a stationary yeast cell desiccated for 14 days, showing a whorl-like structure (boxed and inset) caused by ER stress. (Adopted from reference [12]).

Desiccation stress in stationary yeast cells causes further structural changes [35,36,37]. These changes include altered cell walls, altered cellular and nuclear membranes, and a reduction in cell size. Desiccation also induces oxidative stress, which, in turn, causes DNA and membrane damage [38]. Using transmission electron microscopy (TEM), we recently showed that desiccation in yeast triggers endoplasmic reticulum (ER) stress and unfolded protein response [12]. The ER is misfolded, and the nuclear membrane is ruptured (Figure 2). Vacuoles are not readily observed in desiccated yeast cells. In *C. elegans*, structural damage, including damage to the cell and mitochondrial membrane, is observed in desiccation-sensitive dauers or desiccation-tolerant dauers without proper preconditioning [19].

#### 2.2.2. Physiological Changes

Like structural changes in anhydrobiotes, many physiological changes occur during their preconditioning or the initial low-stress stage. Starvation stress leads to a significant decrease in metabolism within the cells and an increased accumulation of proteins and non-reducing disaccharides that function as stress effectors, including hydrophilins, heat shock proteins, and trehalose. These proteins and non-reducing disaccharides help to preserve both membrane and protein structures as well as to prevent protein misfolding [10,11].

Stress proteins: Stress proteins are composed of three intertwined protein families: heat shock proteins (HSPs), intrinsically disordered proteins (IDPs), and hydrophilins. HSPs are molecular chaperones that contribute to the maintenance of cellular homeostasis by facilitating the refolding of misfolded proteins. They are broadly categorized based on their molecular weights, including Hsp40 (~40 kDa), Hsp60 (57–65 kDa), Hsp70 (68–80 kDa), Hsp90 (83–99 kDa), and small HSPs (15–40 kDa) [39]. HSPs also play crucial roles in stress response. Many HSPs are induced by stress, including heat, osmotic, oxidative, and desiccation stresses [40,41,42]. The yeast small heat shock protein Hsp12p, for example, protects against desiccation stress by interacting with the plasma membrane and protecting the membrane structure during desiccation [43]. The disaggregase Hsp104p also enhances yeast desiccation tolerance. Interestingly, this enhancement is accomplished only in cooperation with the disaccharide trehalose [44]. Hsp70p levels are increased upon stress, but no correlation has been observed between Hsp70p increase and desiccation tolerance in yeast [45]. It would be interesting to see if Hsp70p requires other stress effectors to function. A correlation between the Hsp70p level and desiccation has also been reported in other organisms. In the green algae *Klebsormidium*, the *Hsp70* gene is upregulated in desiccation-tolerant strains upon desiccation stress [27]. In the brine shrimp *Artemia*, knockdown of the *Hsp70* gene reduces the viability of desiccated cysts [46].

Intrinsically disordered proteins (IDPs) are a large and functionally important family of proteins characterized by their lack of stable three-dimensional structures and existence in multiple interconverting conformational states. Many IDPs can change to a fixed tertiary structure after binding to other proteins or RNA [47]. IDPs play important roles in many biological processes, including cell organization, development, and stress tolerance [10,48]. The tardigrade-specific IDPs are essential and sufficient for desiccation tolerance [10]. The late embryogenesis abundant (LEA) proteins are plant-specific IDPs that facilitate the stabilization of membranes during desiccation, enhancing the desiccation tolerance of plant seeds [49]. In yeast, IDP Hsp12p interacts with the plasma membrane and protects membrane structures against desiccation stress [43]. Hsp12p also synergizes with trehalose to mitigate protein aggregation and desiccation stress [11].

Hydrophilins are a group of proteins characterized by a high glycine content (>6%) and high hydrophilicity index (>1.0) [50,51]. Hydrophilins often include intrinsically disordered proteins (IDPs), partially due to their high content of glycine, as a disorder-promoting residue [11]. Genes encoding hydrophilins are induced by osmotic stress, suggesting that these proteins play a role in response to water loss or desiccation stress. Twelve hydrophilin proteins have been identified in *Saccharomyces cerevisiae*. Their possible involvement in desiccation stress is listed in Table 1. While the molecular mechanism in the stress protection of many hydrophilins is not well studied, it is believed that they act to stabilize proteins and membranes during desiccation [52]. Studies have shown that both Sip18p [51] and Stf2p [52] enhance desiccation tolerance by reducing the ROS level during desiccation stress.

Proteins that are not HSPs, IDPs, or hydrophilins may also be involved in membrane stability. The cortical ER protein Ist2p, for example, connects ER to the plasma membrane and stabilizes the membrane during desiccation stress. Ist2p also plays a key role in sustaining or restoring the plasma membrane structure during rehydration [53].

**Table 1:** Yeast hydrophilins and their possible roles in desiccation stress.

Gene ID	Gene Name	Possible Role in (Desiccation) Resistance	References
YJL184W	*GON7*	Involved in cell wall mannoprotein biosynthesis and osmotic stress response.	[54]
YMR175W	*SIP18*	Involved in osmotic and desiccation stress response.	[55,56]
YMR260C	*TIF11*	Localizes to the cytoplasmic stress granule; desiccation resistance increases in the mutant.	[8,57]
YFL014W	*HSP12*	Plant LEA-like protein involved in plasma membrane organization and responses to multiple stresses, including desiccation stress.	[43]
YDL213C	*NOP6*	Required for desiccation–rehydration process.	[52]
YGR008C	*STF2*	Involved in cellular response to desiccation, oxidation, and DNA replication stress.	[52]
YBR016W	*CPP1*	Involved in the adaptive response to hyperosmotic stress. Detailed biological function unknown.	[58]
YPL223C	*GRE1*	Paralog to SIP18. Involved in response to multiple stresses, including osmotic, oxidative, heat shock, and desiccation.	[59]
YFL010C	*WWM1*	Biological function unknown. Interacts with the caspase-related protease Mca1p.	[60]
YJL144W	*ROQ1*	Regulator of the Ubr1p E3 ubiquitin ligase; involved in osmotic, DNA replication, and desiccation stress.	[60,61]
YNL162W	*RPL42A*	Subunit of the large ribosomal 60S subunit. Its function in desiccation stress is unknown.	[52,62]
YNL190W		Cell wall protein, essential for desiccation stress response.	[52]

Trehalose: Trehalose is one of the most well-studied stress effectors in desiccation biology. A study showed that both the structure and the dynamics of the dehydrated matrices differ significantly between trehalose (a non-reducing disaccharide) and sucrose (a reducing disaccharide). The dehydrated trehalose matrix is homogeneous, whereas dehydrated sucrose forms a heterogeneous matrix. This unique structure makes trehalose a more effective stabilizer for proteins under stress [63,64].

The high concentration of trehalose is found in many anhydrobiotes [65]. In budding yeast, elevated levels of trehalose are found only in stationary cells, which are desiccation-tolerant. The trehalose concentration is very low in exponentially growing cells, and these cells are very sensitive to desiccation [22,24]. Stationary yeast cells have a metabolic mode distinct from the exponentially growing cells. Cells growing exponentially use fermentative glycolysis to meet their energetic and biosynthetic needs. When glucose is depleted, cells are transitioned to respiratory metabolism, primarily using ethanol as their energy source [66].

In yeast, both glycolytic and respiratory activities are very low during the stationary phase when trehalose has accumulated [67]. The glyoxylate shunt, or glyoxylate cycle, plays an essential role in trehalose synthesis in both stationary yeast cells and the dauer larva of *C. elegans* [68]. The glyoxylate shunt works by bypassing the steps in the citric acid cycle to produce trehalose using ethanol or acetate as its carbon source. Fatty acids, which are abundant in stationary yeast, could also be used as a carbon source of the glyoxylate cycle [68,69].

Trehalose often cooperates with other stress proteins to establish desiccation tolerance. In yeast, for example, trehalose works together with Hsp12p to promote both short-term and long-term desiccation tolerance [11]. It has also been shown that trehalose cooperates with Hsp104p to promote short-term desiccation tolerance, while trehalose is responsible for long-term survival [44]. In tardigrade, while trehalose levels are relatively low, the effector still plays a crucial role in promoting desiccation tolerance by working synergistically with the tardigrade-specific disordered protein CAHS D [21].

Glycerol: Loss of water alters the osmolarity of cells and, therefore, induces osmotic stress. The high osmolarity glycerol (HOG) pathway is activated upon osmotic stress and increases the production of glycerol, which is the main osmolyte in yeast [70]. However, studies have shown that glycerol does not affect desiccation tolerance [22,71]. More studies are needed to elucidate the possible roles of glycerol in the desiccation stress response.

Proline: Proline is an amino acid that is unique among the standard amino acids in that it does not have a free α-amino group. In bacteria or plants, proline accumulates in response to osmotic stress and functions as an osmoprotectant [72]. In yeast, increasing the proline level by either deleting proline-oxidase (*PUT1*) or externally adding proline enhances both freezing and desiccation resistance [73]. However, in yeast, no proline increase was observed upon stress, unlike in bacteria or plants [72]. Like glycerol, the involvement of proline in desiccation stress warrants further investigation.

## 3. How Do Anhydrobiotes Survive in Desiccated State—Minimal Metabolism but Not Ametabolism

One pressing question is how anhydrobiotes survive during desiccation stress and within the desiccated state. When transitioning from the starvation to desiccation stage, yeast cells lose the two most important ingredients for life: food and water. To counter this problem, cells utilize two strategies–reduce their metabolic activities and use their own food “reservoirs”. When starved, yeast cells accumulate lipid droplets and trehalose to use as an energy reservoir [33]. When they are re-introduced to fresh nutrients, both lipids and trehalose are quickly consumed before cells use external nutrients for cellular growth [34,44]. If stress continues or becomes more severe (starvation plus desiccation), cells may use this reserved energy to further prepare themselves for the anhydrobiotic stage.

**Figure 3:**
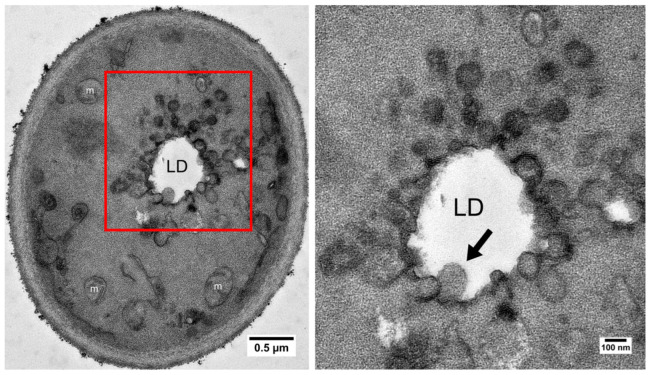
TEM image of a stationary yeast cell desiccated for 14 days, showing the lipid droplet surrounded by circular ER structures. Some of the circular structures appear to have no membrane (arrow in enlarged box area) (Adopted from reference [12]).

The anhydrobiotic state is sometimes considered as anhydrobiotic organisms being in a state of ametabolism or suspended metabolism due to desiccation [74]. In anhydrobiotic nematodes, metabolism cannot be detected using radiolabeled glucose [3]. However, molecular mobility and enzyme activities, although minimal, have been detected in desiccated lichens [75]. Enzyme activity was also observed in desiccated yeast for up to half a year [44]. Trehalose was slowly digested by trehalases while yeast was in its desiccated state. The consumption of trehalose likely provides energy to maintain life. However, the knockout of trehalase genes (*ATH1* and *NTH1*) extends viability after desiccation [44]. It is possible that, in the absence of trehalases, other forms of energy are used, while trehalose is used to preserve the protein and membrane structure. In our recent study, we observed that vacuoles, serving as the primary site for protein degradation and recycling, were mostly diminished 14 days after desiccation [12], suggesting that yeast cells likely use resources recycled from vacuoles to prepare/sustain them for the anhydrobiotic state. We also observed dynamic changes in lipid bodies. The circular membrane structure, induced by desiccation, is merged and releases the lipid into the lipid body (Figure 3); then, the lipid body steadily decreases, likely consumed by cells as an energy source. Defects in lipid droplet synthesis significantly reduce desiccation tolerance, further suggesting that lipid consumption is important for the survival of desiccation.

When compared the gene expression profiles between non-desiccated and desiccated stationary yeast cells, the fatty acid oxidation pathway was highly enriched ([76], Table 2). These data strongly suggest that fatty acid oxidation (β-oxidation) plays a significant role in the desiccation response.

**Table 2:** Desiccation-induced upregulated genes enriched in the fatty acid oxidation pathway in budding yeast.

Gene ID	Gene Name	Description
YER015W	*LPX1*	Peroxisomal matrix-localized lipase: required for normal peroxisomal morphology.
YGL205W	*POX1*	Fatty-acyl coenzyme A oxidase: involved in the fatty acid beta-oxidation pathway.
YIL160C	*POT1*	3-ketoacyl-CoA thiolase with broad chain length specificity: cleaves 3-ketoacyl-CoA into acyl-CoA and acetyl-CoA during beta-oxidation of fatty acids.
YKR009C	*FOX2*	3-hydroxyacyl-CoA dehydrogenase and enoyl-CoA hydratase: multifunctional enzyme of the peroxisomal fatty acid beta-oxidation pathway.
YLR284C	*ECI1*	Peroxisomal delta3, delta2-enoyl-CoA isomerase: essential for the beta-oxidation of unsaturated fatty acids.
YOR180C	*DCI1*	Peroxisomal protein: involved in fatty acid metabolism.

The β-oxidation of fatty acids provides not only necessary energy to cells but also metabolic water. In this process, the fatty acids are broken down into shortened acryl-CoAs and acetyl-CoAs. The acetyl-CoAs are then fully oxidized in mitochondria via the citric acid cycle and oxidative phosphorylation, producing ATP, carbon dioxide, and water [77]. The oxidation of fat produces about 110 g of metabolic water per 100 g of fat, which is a far greater amount than the oxidation of carbohydrates or proteins produces [78,79]. This metabolic water could potentially be crucial to the survival of the desiccated yeast and essential for enzymatic activities [44]. Similarly, metabolic water provides an important water source for animals living in desert landscapes or for migrating birds [80,81].

## 4. Desiccated Yeast as a Model to Study Prion and Other Neurodegenerative Disorders

Yeast has been used as a model system to study many biological aspects of higher eukaryotes [82,83]. It has also been used to tackle many human diseases, including neurodegenerative disorders, such as Parkinson’s disease [84,85]. Desiccation induces protein misfolding, which is a major cause of many neurodegenerative disorders [86]. This could make desiccated yeast good models to study neurodegenerative disorders, such as prion diseases.

Prion diseases are rare yet fatal infections caused by the misfolding and aggregation of proteins known as prions. These protein aggregates cluster within the body, specifically in the brain, and can cause the degeneration and damage of brain tissue [87], often resulting in the hindrance of thinking and reasoning. One of the most common prion diseases in humans is Creutzfeldt-Jakob disease (CJD) [87]. CJD causes the deterioration of brain tissue from the aggregation of prions, inhibiting the normal function of the brain. Currently, there is no known cause for CJD, but there is speculation that the cause is wide-ranging [88].

Budding yeast possesses several prion proteins. They are not homologous to mammal prions but share significant similarities to the amino acid composition and transmission of the phenotype [14,89]. The most studied yeast prion is [PSI^+^], a misfolded aggregate of the Sup35 protein. Sup35 is a translation termination factor from the eRF3 family [90]. An easy way to identify [PSI^+^] formation is using an ADE2 mutant strain that contains a premature stop codon. In the absence of [PSI^+^], i.e., [psi^−^], the colony of this strain becomes red in a rich medium that lacks additional adenine. When the yeast cells contain [PSI^+^] positive proteins, the colonies become white due to the misfolded, non-functional Sup35 skipping the premature stop codon, producing a full-length Ade2 protein [91]. Using this strategy, we isolated and examined the [PSI^+^] strain with and without desiccation stress using TEM. Our preliminary results showed that heavy [PSI^+^] aggregates were formed under desiccation, suggesting the feasibility of using desiccation stress to study prion biology.

## 5. Concluding Remarks

As an anhydrobiote, budding yeast is highly tolerant to water loss, providing us with a window to understand desiccation stress and stress response. Due to the complex nature of desiccation stress, more studies are necessary to unravel the molecular mechanisms that accompany this type of stress, especially since desiccation stress interconnects with other stresses, such as osmotic or oxidative stress. Special focus should be given to the interplay between different stresses and different stress effectors on the same type of stress. Another point of interest that is less studied is the rehydration process, which could also cause multiple stresses to the organism. The outcomes of these studies will not only further our understanding of stress biology but also pave the way for more groundbreaking medical applications, such as using desiccation technology as an alternative for the storage of biological materials like vaccines [92].

## References

[1] Dargaville B.L., Hutmacher D.W. (2022). Water as the often neglected medium at the interface between materials and biology. Nat. Commun..

[2] Cowan N.B., Agol E., Meadows V.S., Robinson T., Livengood T.A., Deming D., Lisse C.M., A’Hearn M.F., Wellnitz D.D., Seager S. (2009). Allen maps of an ocean-bearing world. Astrophys. J..

[3] Wharton D.A. (2015). Anhydrobiosis. Curr. Biol..

[4] Goldstein B., Blaxter M. (2002). Tardigrades. Curr. Biol..

[5] Smolikova G., Leonova T., Vashurina N., Frolov A., Medvedev S. (2020). Desiccation tolerance as the basis of long-term seed viability. Int. J. Mol. Sci..

[6] Hesgrove C., Boothby T.C. (2020). The biology of tardigrade disordered proteins in extreme stress tolerance. Cell Commun. Signal..

[7] Rapoport A., Turchetti B., Buzzini P. (2016). Application of anhydrobiosis and dehydration of yeasts for non-conventional biotechnological goals. World J. Microbiol. Biotechnol..

[8] Welch A.Z., Gibney P.A., Botstein D., Koshland D.E. (2013). TOR and RAS pathways regulate desiccation tolerance in *Saccharomyces cerevisiae*. Mol. Biol. Cell.

[9] Gasch A.P., Spellman P.T., Kao C.M., Carmel-Harel O., Eisen M.B., Storz G., Botstein D., Brown P.O. (2000). Genomic expression programs in the response of yeast cells to environmental changes. Mol. Biol. Cell.

[10] Boothby T.C., Tapia H., Brozena A.H., Piszkiewicz S., Smith A.E., Giovannini I., Rebecchi L., Pielak G.J., Koshland D., Goldstein B. (2017). Tardigrades use intrinsically disordered proteins to survive desiccation. Mol. Cell.

[11] Kim S.X., Çamdere G., Hu X., Koshland D., Tapia H. (2018). Synergy between the small intrinsically disordered protein Hsp12 and trehalose sustain viability after severe desiccation. Elife.

[12] Ren Q., Brenner R., Boothby T.C., Zhang Z. (2020). Membrane and lipid metabolism plays an important role in desiccation resistance in the yeast *Saccharomyces cerevisiae*. BMC Microbiol..

[13] Tebele S.M., Marks R.A., Farrant J.M. (2021). Two decades of desiccation biology: A systematic review of the best studied angiosperm resurrection plants. Plants.

[14] Liebman S.W., Chernoff Y.O. (2012). Prions in yeast. Genetics.

[15] Collinson L.P., Dawes I.W. (1992). Inducibility of the response of yeast cells to peroxide stress. J. Gen. Microbiol..

[16] Hoekstra F.A., Golovina E.A., Buitink J. (2001). Mechanisms of plant desiccation tolerance. Trends Plant Sci..

[17] Ingram J., Bartels D. (1996). The molecular basis of dehydration tolerrance in plants. Annu. Rev. Plant Physiol. Plant Mol. Biol..

[18] Crowe J.H., Madin K.A.C. (1975). Anhydrobiosis in nematodes: Evaporative water loss and survival. J. Exp. Zool..

[19] Erkut C., Penkov S., Khesbak H., Vorkel D., Verbavatz J.-M., Fahmy K., Kurzchalia T.V. (2011). Trehalose renders the dauer larva of *Caenorhabditis elegans* resistant to extreme desiccation. Curr. Biol..

[20] Boothby T.C., Tenlen J.R., Smith F.W., Wang J.R., Patanella K.A., Nishimura E.O., Tintori S.C., Li Q., Jones C.D., Yandell M. (2015). Evidence for extensive horizontal gene transfer from the draft genome of a tardigrade. Proc. Natl. Acad. Sci. USA.

[21] Nguyen K., Kc S., Gonzalez T., Tapia H., Boothby T.C. (2022). Trehalose and tardigrade CAHS proteins work synergistically to promote desiccation tolerance. Commun. Biol..

[22] Calahan D., Dunham M., DeSevo C., Koshland D.E. (2011). Genetic analysis of desiccation tolerance in *Sachharomyces cerevisiae*. Genetics.

[23] Huang M., Hull C.M. (2017). Sporulation: How to survive on planet Earth (and beyond). Curr. Genet..

[24] Zhang Z., Zhang G.R. (2022). Chromosome-condensed G1 phase yeast cells are tolerant to desiccation stress. Microb. Cell.

[25] Bagniewska-Zadworna A. (2008). The root microtubule cytoskeleton and cell cycle analysis through desiccation of *Brassica napus* seedlings. Protoplasma.

[26] Kakumanu A., Ambavaram M.M., Klumas C., Krishnan A., Batlang U., Myers E., Grene R., Pereira A. (2012). Effects of drought on gene expression in maize reproductive and leaf meristem tissue revealed by RNA-Seq. Plant Physiol..

[27] Rippin M., Borchhardt N., Karsten U., Becker B. (2019). Cold acclimation improves the desiccation stress resilience of polar strains of *Klebsormidium* (Streptophyta). Front. Microbiol..

[28] Slade D., Radman M. (2011). Oxidative stress resistance in *Deinococcus radiodurans*. Microbiol. Mol. Biol. Rev..

[29] Lew D.J., Weinert T., Pringle J.R., Pringle J.R., Broach J.R., Jones E.W. (1997). Cell cycle control in *Saccharomyces cerevisiae*. The Molecular and Cellular Biology of the Yeast Saccharomyces: Cell Cycle and Cell Biology.

[30] Yang H., Ren Q., Zhang Z. (2008). Cleavage of Mcd1 by caspase-like protease Esp1 promotes apoptosis in budding yeast. Mol. Biol. Cell.

[31] Werner-Washburne M., Braun E., Johnston G.C., Singer R.A. (1993). Stationary phase in the yeast *Saccharomyces cerevisiae*. Microbiol. Rev..

[32] de Nobel H., Ruiz C., Martin H., Morris W., Brul S., Molina M., Klis F.M. (2000). Cell wall perturbation in yeast results in dual phosphorylation of the Slt2/Mpk1 MAP kinase and in an Slt2-mediated increase in FKS2-lacZ expression, glucanase resistance and thermotolerance. Microbiology.

[33] Wolinski H., Kolb D., Hermann S., Koning R.I., Kohlwein S.D. (2011). A role for seipin in lipid droplet dynamics and inheritance in yeast. J. Cell Sci..

[34] Kurat C.F., Natter K., Petschnigg J., Wolinski H., Scheuringer K., Scholz H., Zimmermann R., Leber R., Zechner R., Kohlwein S.D. (2006). Obese yeast: Triglyceride lipolysis is functionally conserved from mammals to yeast. J. Biol. Chem..

[35] Beker M.J., Rapoport A.I. (1987). Conservation of yeasts by dehydration. Biotechnology Methods.

[36] Rapoport A., Sibirny A.A. (2017). Anhydrobiosis and dehydration of yeasts. Biotechnology of Yeasts and Filamentous Fungi.

[37] Rapoport A., Golovina E.A., Gervais P., Dupont S., Beney L. (2019). Anhydrobiosis: Inside yeast cells. Biotechnol. Adv..

[38] Pereira Ede J., Panek A.D., Eleutherio E.C. (2003). Protection against oxidation during dehydration of yeast. Cell Stress. Chaperones.

[39] Hagymasi A.T., Dempsey J.P., Srivastava P.K. (2022). Heat-shock proteins. Curr. Protoc..

[40] Schlesinger M.J. (1990). Heat shock proteins. J. Biol. Chem..

[41] De Maio A. (1999). Heat shock proteins: Facts, thoughts, and dreams. Shock.

[42] Hu C., Yang J., Qi Z., Wu H., Wang B., Zou F., Mei H., Liu J., Wang W., Liu Q. (2022). Heat shock proteins: Biological functions, pathological roles, and therapeutic opportunities. MedComm.

[43] Sales K., Brandt W., Rumbak E., Lindsey G. (2000). The LEA-like protein HSP 12 in *Saccharomyces cerevisiae* has a plasma membrane location and protects membranes against desiccation and ethanol-induced stress. Biochim. Biophys. Acta.

[44] Tapia H., Young L., Fox D., Bertozzi C.R., Koshland D. (2015). Increasing intracellular trehalose is sufficient to confer desiccation tolerance to Saccharomyces cerevisiae. Proc. Natl. Acad. Sci. USA.

[45] Guzhova I., Krallish I., Khroustalyova G., Margulis B., Rapoport A. (2008). Dehydration of yeast: Changes in the intracellular content of Hsp70 family proteins. Process Biochem..

[46] Iryani M.T.M., Sorgeloos P., Danish-Daniel M., Tan M.P., Wong L.L., Mok W.J., Satyantini W.H., Mahasri G., Sung Y.Y. (2020). Cyst viability and stress tolerance upon heat shock protein 70 knockdown in the brine shrimp Artemia franciscana. Cell Stress. Chaperones.

[47] Trivedi R., Nagarajaram H.A. (2022). Intrinsically Disordered Proteins: An Overview. Int. J. Mol. Sci..

[48] Chakrabortee S., Tripathi R., Watson M., Schierle G.S., Kurniawan D.P., Kaminski C.F., Wise M.J., Tunnacliffe A. (2012). Intrinsically disordered proteins as molecular shields. Mol. Biosyst..

[49] Hincha D.K., Thalhammer A. (2012). LEA proteins: IDPs with versatile functions in cellular dehydration tolerance. Biochem. Soc. Trans..

[50] Garay-Arroyo A., Colmenero-Flores J.M., Garciarrubio A., Covarrubias A.A. (2000). Highly hydrophilic proteins in prokaryotes and eukaryotes are common during conditions of water deficit. J. Biol. Chem..

[51] Rodríguez-Porrata B., Carmona-Gutierrez D., Reisenbichler A., Bauer M., Lopez G., Escoté X., Mas A., Madeo F., Cordero-Otero R. (2012). Sip18 hydrophilin prevents yeast cell death during desiccation stress. J. Appl. Microbiol..

[52] López-Martínez G., Rodríguez-Porrata B., Margalef-Català M., Cordero-Otero R. (2012). The STF2p hydrophilin from *Saccharomyces cerevisiae* is required for dehydration stress tolerance. PLoS ONE.

[53] Dauss E., Papoušková K., Sychrová H., Rapoport A. (2021). Anhydrobiosis in yeast: Role of cortical endoplasmic reticulum protein Ist2 in *Saccharomyces cerevisiae* cells during dehydration and subsequent rehydration. Antonie Van Leeuwenhoek.

[54] Ando A., Tanaka F., Murata Y., Takagi H., Shima J. (2006). Identification and classification of genes required for tolerance to high-sucrose stress revealed by genome-wide screening of *Saccharomyces cerevisiae*. FEMS Yeast Res..

[55] Miralles V.J., Serrano R. (1995). A genomic locus in Saccharomyces cerevisiae with four genes up-regulated by osmotic stress. Mol. Microbiol..

[56] Dang N.X., Hincha D.K. (2011). Identification of two hydrophilins that contribute to the desiccation and freezing tolerance of yeast (*Saccharomyces cerevisiae*) cells. Cryobiology.

[57] Jain S., Wheeler J.R., Walters R.W., Agrawal A., Barsic A., Parker R. (2016). ATPase-modulated stress granules contain a diverse proteome and substructure. Cell.

[58] Venancio T.M., Aravind L. (2010). CYSTM, a novel cysteine-rich transmembrane module with a role in stress tolerance across eukaryotes. Bioinformatics.

[59] Garay-Arroyo A., Covarrubias A.A. (1999). Three genes whose expression is induced by stress in Saccharomyces cerevisiae. Yeast.

[60] Szoradi T., Schaeff K., Garcia-Rivera E.M., Itzhak D.N., Schmidt R.M., Bircham P.W., Leiss K., Diaz-Miyar J., Chen V.K., Muzzey D. (2018). SHRED is a regulatory cascade that reprograms Ubr1 substrate specificity for enhanced protein quality control during stress. Mol. Cell.

[61] Tkach J.M., Yimit A., Lee A.Y., Riffle M., Costanzo M., Jaschob D., Hendry J.A., Ou J., Moffat J., Boone C. (2012). Dissecting DNA damage response pathways by analysing protein localization and abundance changes during DNA replication stress. Nat. Cell Biol..

[62] Planta R.J., Mager W.H. (1998). The list of cytoplasmic ribosomal proteins of *Saccharomyces cerevisiae*. Yeast.

[63] Jain N.K., Roy I. (2009). Effect of trehalose on protein structure. Protein Sci..

[64] Malferrari M., Nalepa A., Venturoli G., Francia F., Lubitz W., Möbius K., Savitsky A. (2014). Structural and dynamical characteristics of trehalose and sucrose matrices at different hydration levels as probed by FTIR and high-field EPR. Phys. Chem. Chem. Phys..

[65] Crowe J.H. (2007). Trehalose as a “chemical chaperone”: Fact and fantasy. Adv. Exp. Med. Biol..

[66] Dickinson J.R., Schweizer M. (2004). Metabolism and Molecular Physiology of Saccharomyces Cerevisiae.

[67] François J., Parrou J.L. (2001). Reserve carbohydrates metabolism in the yeast *Saccharomyces cerevisiae*. FEMS Microbiol. Rev..

[68] Erkut C., Gade V.R., Laxman S., Kurzchalia T.V. (2016). The glyoxylate shunt is essential for desiccation tolerance in *C. elegans* and budding yeast. Elife.

[69] Ahn S., Jung J., Jang I.A., Madsen E.L., Park W. (2016). Role of glyoxylate shunt in oxidative stress response. J. Biol. Chem..

[70] de Nadal E., Posas F. (2022). The HOG pathway and the regulation of osmoadaptive responses in yeast. FEMS Yeast Res..

[71] Semkiv M., Ternavska O.T., Dmytruk K.V., Sybirny A.A. (2018). Effect of trehalose and glycerol on the resistance of recombinant *Saccharomyces cerevisiae* Strains to desiccation, freeze-thaw and osmotic stresses. Sci. Innov..

[72] Takagi H. (2008). Proline as a stress protectant in yeast: Physiological functions, metabolic regulations, and biotechnological applications. Appl. Microbiol. Biotechnol..

[73] Takagi H., Sakai K., Morida K., Nakamori S. (2000). Proline accumulation by mutation or disruption of the proline oxidase gene improves resistance to freezing and desiccation stresses in *Saccharomyces cerevisiae*. FEMS Microbiol. Lett..

[74] Grzyb T., Skłodowska A. (2022). Introduction to Bacterial Anhydrobiosis: A General perspective and the mechanisms of desiccation-associated damage. Microorganisms.

[75] Candotto Carniel F., Fernandez-Marín B., Arc E., Craighero T., Laza J.M., Incerti G., Tretiach M., Kranner I. (2021). How dry is dry? Molecular mobility in relation to thallus water content in a lichen. J. Exp. Bot..

[76] Singh J., Kumar D., Ramakrishnan N., Singhal V., Jervis J., Garst J.F., Slaughter S.M., DeSantis A.M., Potts M., Helm R.F. (2005). Transcriptional response of Saccharomyces cerevisiae to desiccation and rehydration. Appl Environ Microbiol.

[77] Wanders R.J., Waterham H.R., Ferdinandusse S. (2015). Metabolic Interplay between Peroxisomes and Other Subcellular Organelles Including Mitochondria and the Endoplasmic Reticulum. Front. Cell Dev. Biol..

[78] Mellanby K. (1942). Metabolic Water and Desiccation. Nature.

[79] Schmidt-Nielsen K. (1997). Animal Physiology: Adaptation and Environment.

[80] Klaassen M. (1996). Metabolic constraints on long-distance migration in birds. J. Exp. Biol..

[81] Takei Y. (2024). Metabolic Water As a Route for Water Acquisition in Vertebrates Inhabiting Dehydrating Environments. Zool. Sci..

[82] Locascio A., Andrés-Colás N., Mulet J.M., Yenush L. (2019). Saccharomyces cerevisiae as a Tool to Investigate Plant Potassium and Sodium Transporters. Int. J. Mol. Sci..

[83] Vanderwaeren L., Dok R., Voordeckers K., Nuyts S., Verstrepen K.J. (2022). *Saccharomyces cerevisiae* as a model system for eukaryotic cell biology, from cell cycle control to DNA damage response. Int. J. Mol. Sci..

[84] Wang S., Xu B., Liou L.C., Ren Q., Huang S., Luo Y., Zhang Z., Witt S.N. (2012). α-Synuclein disrupts stress signaling by inhibiting polo-like kinase Cdc5/Plk2. Proc. Natl. Acad. Sci. USA.

[85] Witt S.N., Flower T.R. (2006). alpha-Synuclein, oxidative stress and apoptosis from the perspective of a yeast model of Parkinson’s disease. FEMS Yeast Res..

[86] Ross C.A., Poirier M.A. (2004). Protein aggregation and neurodegenerative disease. Nat. Med..

[87] Soto C., Satani N. (2011). The intricate mechanisms of neurodegeneration in prion diseases. Trends Mol. Med..

[88] Rossi M., Baiardi S., Parchi P. (2019). Understanding prion strains: Evidence from studies of the disease forms affecting humans. Viruses.

[89] Wickner R.B. (1994). [URE3] as an altered URE2 protein: Evidence for a prion analog in *Saccharomyces cerevisiae*. Science.

[90] Kushnirov V.V., Kochneva-Pervukhova N.V., Chechenova M.B., Frolova N.S., Ter-Avanesyan M.D. (2000). Prion properties of the Sup35 protein of yeast Pichia methanolica. EMBO J..

[91] Derkatch I.L., Chernoff Y.O., Kushnirov V.V., Inge-Vechtomov S.G., Liebman S.W. (1996). Genesis and variability of [PSI] prion factors in Saccharomyces cerevisiae. Genetics.

[92] Worrall E.E., Litamoi J.K., Seck B.M., Ayelet G. (2000). Xerovac: An ultra rapid method for the dehydration and preservation of live attenuated Rinderpest and Peste des Petits ruminants vaccines. Vaccine.

